# Synchronized Orchestration of miR-99b and let-7g Positively Regulates Rotavirus Infection by Modulating Autophagy

**DOI:** 10.1038/s41598-018-38473-8

**Published:** 2019-02-04

**Authors:** Urbi Mukhopadhyay, Shampa Chanda, Upayan Patra, Arpita Mukherjee, Santanu Rana, Anupam Mukherjee, Mamta Chawla-Sarkar

**Affiliations:** 1 Division of Virology, National Institute of Cholera and Enteric Diseases, Kolkata, WB India; 2 Department of Zoology, University of Calcutta, Kolkata, WB India

## Abstract

Rotavirus (RV), the major etiological agent of viral gastroenteritis in young children, kills over 200 thousand infants each year. In spite of available vaccines, rotaviral diarrhoea is still a major problem in developing countries of Asia and Africa. Therefore, the studies on RV infection and host antiviral responses are warranted. The active correlation between virus infection and activation of autophagy machinery and positive influence of autophagy on RV replication have been documented recently. Previous study from our group showed dysregulation of several cellular miRNAs during RV infection, though their significance remained largely unknown. Since cellular microRNAs (miRNAs) have been implicated in the control of several fundamental biological processes including stress response and autophagy, we focused on two miRNAs, miR-99b and let-7g, and analyzed their function to gain insight into the miRNA-autophagy crosstalk during RV infection. This study shows that RV suppresses let-7g expression but enhances miR-99b that in turn augment major autophagy regulators. Ectopic expression of let-7g and knockdown of miR-99b resulted in inhibition of autophagy, hence, reduction of RV replication. Overall, our study highlights new mechanistic insights for understanding the role of miRNAs in modulating RV infection and possibility of using RNA interference as an antiviral therapeutic target.

## Introduction

MicroRNAs (miRNAs) are evolutionary conserved, single-stranded, small non-coding RNA molecules that bind to the target mRNA through specific base-pairing interactions between the “seed” region of miRNA and sites within coding and untranslated regions (UTRs) especially 3′UTR of mRNAs to suppress gene expression either by mRNA degradation or translational repression^1^. Dysregulation of miRNAs have been associated with a number of diseases including cardiovascular diseases^2^, malignancies^3^, skin diseases^4^, and autoimmune diseases^5^. Understanding the central role of miRNAs in disease regulation has provided an innovative perspective and offered new therapeutic modalities^6^. Similarly, studying differences in miRNA expression in host cells after virus infection would contribute to our understanding of the viral pathogenesis. Viral infection can exert a profound impact on the cellular miRNA expression profile as reported in hepatitis C virus (HCV), herpesviruses, retroviruses, hepatitis B virus (HBV) etc^7,8^. Given the importance and adaptability of miRNAs, many viruses exploit the host cellular mechanisms by destroying, boosting, or hijacking miRNAs to promote their own stability and propagation^8^. Human miR-122, miR-130a, and miR-373 have been shown to functionally augment hepatitis C virus (HCV) replication, while several other miRNAs, including miR-125b, miR-181c, miR-199a-3p, and miR-323, are found to repress human immunodeficiency virus (HIV), HCV, HBV and Influenza virus replication^9–14^. Unfortunately, there are very limited reports available on the role of miRNAs in regulating rotavirus infection by modulation of host cell responses.

Rotavirus (RV), a non-enveloped double-stranded RNA virus of *Reoviridae* family, is one of the major cause of infantile gastroenteritis and childhood mortality worldwide^15^. RV, like all other RNA viruses, establishes a complex interaction with the host signalling pathways to take advantage of cellular processes for their own survival and replication^16^. Changes in miRNA expression profile during rotavirus infection have recently been studied^17,18^. The previous study from our group has identified sixteen differentially regulated miRNAs during RV infection and showed the pro-viral function of hsa-miR-142-5p by modulation of TGF-β-induced non-canonical signalling^17^. Another study reported the antiviral role of mml-miR-7 and mml-miR-125a during early hours of RV infection^18^. Further, in-depth analysis of microRNAs in controlling different cellular processes to promote or inhibit RV replication will lead to a better understanding of viral pathogenesis.

Emerging line of evidence suggests that miRNAs are closely linked to virtually all known fundamental biological pathways like stress response, proliferation, differentiation, apoptosis, autophagy etc^8,19,20^. Cooperative interactions between multiple microRNAs regulating multiple targets result in an additive effect on many important biological processes^21–23^. Autophagy is a tightly regulated catabolic process, which plays an essential role in maintaining cellular homeostasis and restriction of pathogen replication^24^. Macroautophagy involves the formation of double-membrane-bound vesicles called autophagosomes that engulf cytoplasmic proteins and organelles; these autophagosomes are trafficked to lysosomes for degradation^24,25^. The physiological significance of miRNA-autophagy interconnection in human diseases such as cancer and cardiovascular diseases has been documented in recent years^26,27^. The first link established between miRNAs and autophagy showed that miR-30a directly targets Beclin-1 resulting in decreased autophagic activity in cancer cells^28^. miR-101 is reported to target STMN1, RAB5A, and ATG4D to inhibit autophagy in breast cancer cells and miR-204 blocks cardiomyocyte autophagy by modulating the levels of LC3II^29,30^. Cellular stress conditions, such as virus infection or nutrient deficiency, rapidly activate autophagy and affect the survival of virus-infected or transformed cells^20,24,25^.

As viruses are obligate intracellular parasites, their survival is intricately associated with their ability to regulate cellular processes promoting viral replication as well as in subverting cellular defence mechanisms. Recent studies show that despite the ability of autophagy to act as an antiviral mechanism, some viruses use the autophagy machinery in favour of viral replication^31^. Influenza A virus and Flavivirus NS4A induce autophagy to control cell death and therefore enhancing viral replication^32,33^. Previous studies have shown that RV-NSP4 induces early stages of autophagy by activating CaMKK-β and AMPK-dependent signalling pathway to facilitate the transportation of viral proteins from the endoplasmic reticulum to viroplasm for production of infectious virus particle^24,34^. miRNAs are known to play a significant role in the regulation of autophagy in many virus infections. Recently, miR-141 has been reported to target Sirt1 to inhibit autophagy, whereas, miRNA-99 family was reported to promote autophagy through IGF1R-AKT-mTOR-ULK1 signalling during HBV infection^35,36^. In spite of reports on RV induced autophagy, the role of miRNA-mediated regulation of autophagy during RV life cycle remains completely unexplored.

In the present study, we have analyzed the regulation of autophagy by coordinated expression of cellular microRNAs following RV infection. From the miRNA microarray data, *in silico* analysis was done to identify the microRNAs targeting autophagy signalling pathways. Two miRNAs namely miR-99b and let-7g were identified as potential modulator of autophagy via TSC1/2-mTOR pathway. RV infection causes significant downregulation of let-7g to stabilize the TSC1-TSC2, that resulting in the inhibition of mTOR activity. In addition, following RV infection, miR-99b is upregulated and directly targets mTOR. Exogenous expression of let-7g and knockdown of miR-99b, individually or in combination, restricts RV replication by inhibiting autophagy. This study indicates that following virus infection, myriads of cellular signalling pathways act in coordination to facilitate or antagonize virus infection, e.g. here we report that both the RV-NSP4 regulated CaMKK-β pathway^24^ and the cellular microRNAs synergistically regulate the autophagy pathway to facilitate RV infection.

## Results

### RV infection upregulates miR-99b to aid viral replication

**Figure 1:**
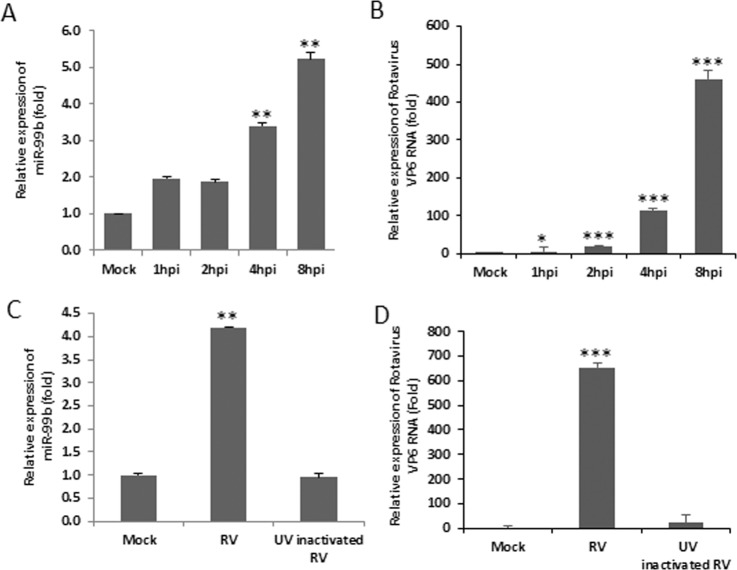
RV infection upregulates miR-99b. HT29 cells were mock treated or infected with RV-SA11 for indicated time points. Total RNA from mock- or virus-infected cells was extracted. (**A**) Expression of miR-99b was analyzed in RV-infected HT29 cells by qRT-PCR and normalized to the expression of U6 snRNA. (**B**) RV-VP6 RNA level was measured in SA11 infected HT29 cell lysate by qRT-PCR and plotted as relative RNA level in comparison to mock-infected cells. (**C**) Relative expression of miR-99b was analyzed by qRT-PCR in UV inactivated RV-SA11 infected (8hpi) HT29 cells. (**D**) RV-VP6 RNA was measured by qRT-PCR in UV-inactivated RV-SA11 infected cells. Results are presented as the means and standard deviations from at least three independent experiments.

The functions of miRNAs vary under different pathophysiological conditions as well as cell types. RV infects and damages the enterocytes, simple columnar epithelial cells, which line the small intestine. Therefore, a miRNA microarray was performed using RNA from mock- or RV-infected human epithelial HT29 cells^17^. miR-99b was one of the significantly upregulated miRNAs in RV infected cells (Supplementary Fig. S1A)^17^. We did an *in silico* analysis using the mimiRNA database to check the abundance of miR-99b in HT29 cells and small intestinal tissues. miR-99b was found to be abundant in HT29 cells as well as in small intestinal tissues with standard score of ~600 (P < 0.01) and ~480 (P < 0.01) respectively (Supplementary Fig. S1B). Next, we validated the upregulation of miR-99b in two different cell lines (HT29 and MA104) infected with RV strain SA11 and KU at different hours post infection (hpi) by qRT-PCR, respectively (Figs 1A and S1C). Expression of VP6 transcript was also analysed in the same samples for confirmation of virus infection (Figs 1B and S1D). However, no change was found in the expression of miR-99b when HT29 cells were infected with the same MOI of UV-inactivated RV-SA11, indicating RV-specific regulation of miR-99b (Fig. 1C). Indeed, expression of VP6 RNA was also negligible in UV-inactivated RV infected cells compared to RV-SA11 infected cells (Fig. 1D). As control for virus infection, canine kidney epithelial cell line MDCK was infected with Influenza A/PR8 strain. Unlike RV-SA11 infection, InfA/PR8 infected cells did not show any change in miR-99b expression (Supplementary Fig. S1E).

**Figure 2:**
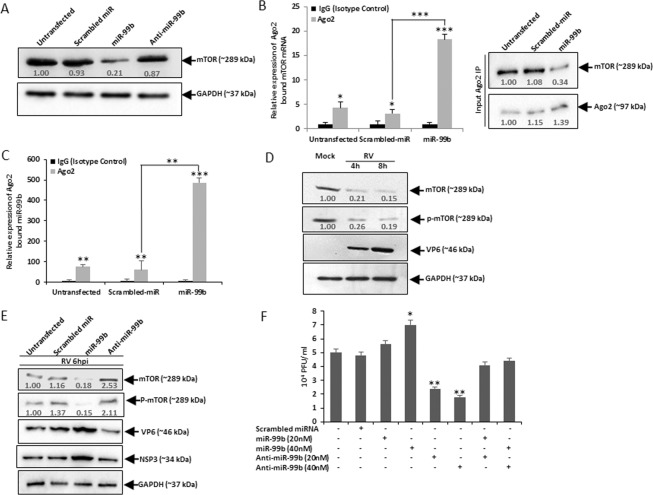
miR-99b controls RV replication by directly targeting mTOR. (**A**) HT29 cells were transfected with scrambled-miR or mimic/anti-miR-99b (40 nM) followed by western blot analysis using specific antibody against mTOR. Relative fold change was determined after normalization to GAPDH. (**B**) Lysed cell extracts either transfected with scrambled-miR or miR-99b (40 nM), were immunoprecipitated with anti-Ago2 antibody or isotype control i.e. rabbit IgG2a; following elution from beads. RNA was isolated and miR-99b bound mTOR mRNA expression was analyzed by qRT-PCR (left panel). mTOR and Ago2 expressions were analysed in the input lysates kept from each sample (right panel). Relative expression was determined after normalization to control cells. (**C**) Relative expression of miR-99b was determined in Ago2 immunoprecipitates by qRT-PCR. (**D**) Cells were mock infected or infected with RV followed by immunoblot analysis. Relative expression of mTOR, p-mTOR, and RV-VP6 was determined using specific antibodies. (**E**) HT29 cells were mock transfected or transfected with scrambled-miR or mimic/anti-miR-99b (40 nM) followed by RV-SA11 infection (6hpi); whole cell lysates were prepared, followed by immunoblotting analysis against mTOR, p-mTOR, and RV-VP6. Fold changes were measured after normalization with GAPDH. (**F**) HT29 cells were transfected with mimic/anti-miR-99b followed by RV-SA11 infection (6hpi) and viral titer was measured by plaque assay. All results are presented as the means and standard deviations from three independent experiments.

To understand the consequence of RV mediated upregulation of miR-99b, *in silico* analysis was done to identify the potential targets using microRNA.org and miRBase algorithms. The results revealed mTOR as a direct target of miR-99b (Supplementary Fig. S1F). To examine the role of miR-99b in modulation of mTOR expression, HT29 cells were transfected with either mimic miR-99b or anti-miR-99b. Significant downregulation of mTOR expression (4.8 fold) was observed in presence of mimic miR-99b by immunoblot analysis (Fig. 2A). For detailed analysis, association of miR-99b with mTOR was examined in HT29 cells by RNA immunoprecipitation (RIP) to isolate Ago2 bound mTOR mRNA. The expression of Ago2 bound mTOR mRNA significantly increased in lysed cell extracts of miR-99b overexpressing cells pulled down with anti-Ago2 antibody compared to the isotype control (Fig. 2B). Furthermore, Ago2 bound mTOR mRNA expression was much higher in mimic miR-99b transfected Ago2 immunoprecipitates compared to scrambled-miR transfected samples (Fig. 2B), confirming the direct association between miR-99b and mTOR. To examine the interaction between miR-99b-mTOR during RV infection, HT29 cells were transfected with scrambled miR or anti-miR-99b followed by RV infection and Ago2 RIP was done (Supplementary Fig. S1G). Induction in Ago2 bound mTOR mRNA driven by RV mediated upregulation of miR-99b was reversed in anti-miR-99b transfected Ago2 immunoprecipitates. Expressions of Ago2 and mTOR proteins were checked in the input lysates (Figs 2B and S1G). Transfection efficiency of mimic miR-99b was confirmed by qRT-PCR in Ago2-immunoprecipitates (Fig. 2C).

Previous studies have suggested that autophagy is a major regulator of RV infection and RV promotes autophagosome formation for proper viral replication^24^. mTOR is known to be a major negative regulator of autophagy^37^. Therefore, we examined the expression levels of mTOR and p-mTOR during early hours of RV infection. Significant downregulation of mTOR expression (5–7 fold) was found at 4–8 hpi (Fig. 2D). The expression of mTOR was also determined in miR-99b overexpressed or knockdown cells infected with RV. miR-99b mimic downregulated the expression of total mTOR and p-mTOR but the presence of anti-miR-99b significantly upregulated its expression, whereas, scrambled miR had no effect on mTOR expression (Fig. 2E).

To understand the importance of miR-99b during infection, RV-SA11 infected HT29 cells were transfected with either anti-miR-99b or mimic miR-99b in a dose-dependent manner and viral titre was measured by plaque assay. Results showed that overexpression of miR-99b resulted in increased viral replication whereas knockdown of miR-99b led to a decrease in RV infection compared to scrambled miRNA (Fig. 2F). Furthermore, ectopic expression of anti-miR-99b in miR-99b overexpressed RV infected cells partially restored the viral replication level as evident in plaque assay (Fig. 2F). These results suggest that miR-99b modulates the expression of mTOR following RV infection and promotes viral replication.

### RV infection suppresses let-7g to promote TSC1

**Figure 3:**
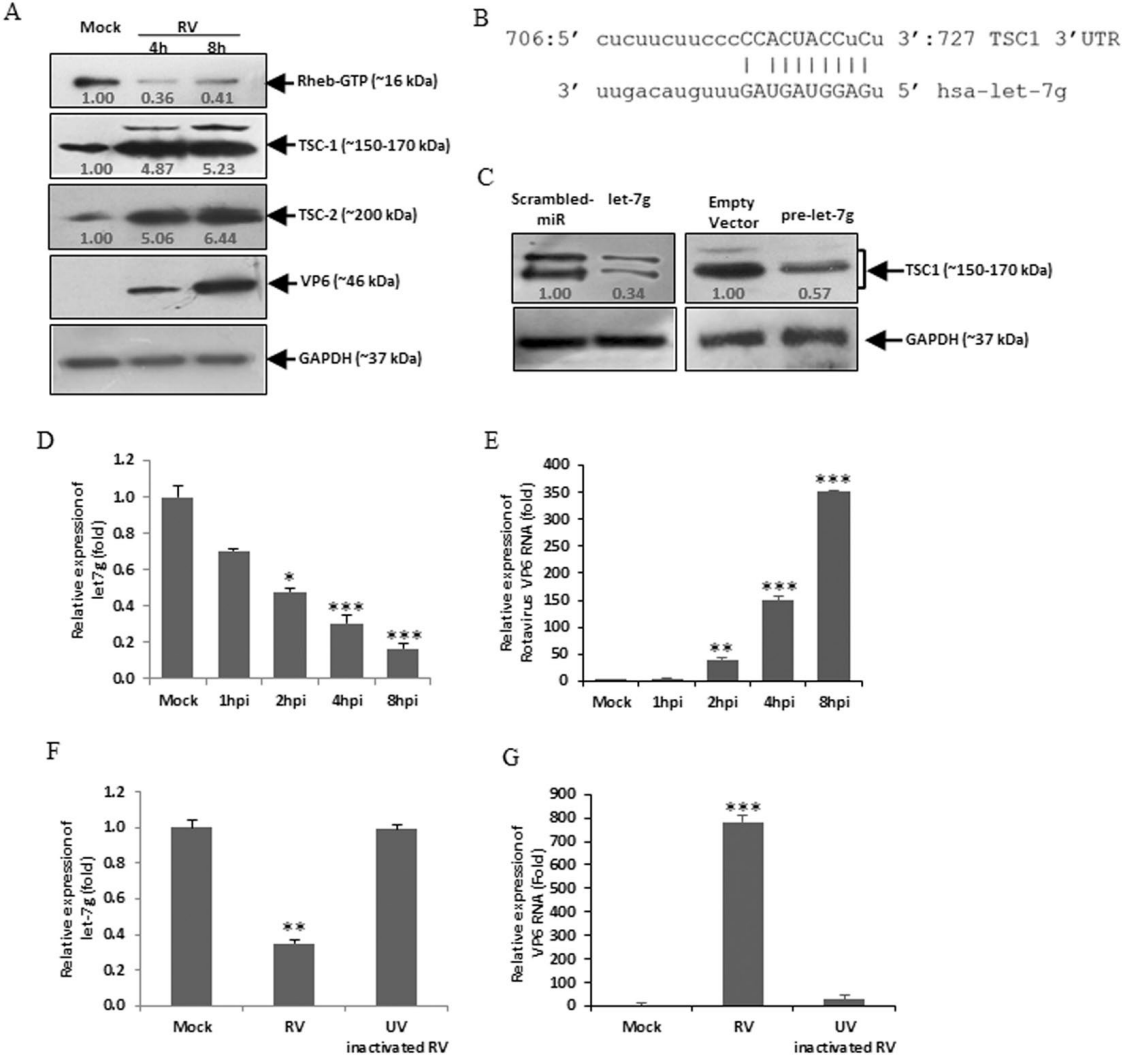
RV infection downregulates let-7g expression. (**A**) HT29 cells were mock treated or infected with RV for indicated time points. Whole cell lysates were prepared, followed by western blot analysis using specific antibodies against Rheb-GTP, TSC1, TSC2, and RV-VP6. The blot was reprobed with an antibody to GAPDH for normalization. (**B**) *In silico* analysis of the TSC1 3′UTR revealed a single putative let-7g binding site. The let-7g target region of TSC1 (GenBank accession number NM_000368) is indicated. (**C**) HT29 cells were transfected with mimic let-7g (40 nM) (left panel) or pmR-ZsGreen1-pre-let7g (right panel) followed by western blot analysis using specific antibody against TSC1. Relative fold differences of the TSC1 level was analyzed after normalization with GAPDH from at least three independent experiments. (**D**) HT29 cells were mock treated or infected with RV-SA11 for indicated time points. Total RNA from mock- or virus-infected cells was extracted and expression of let-7g was analyzed by qRT-PCR and normalized to the expression of U6 snRNA. (**E**) RV-VP6 RNA level was measured and plotted as relative RNA level in comparison to mock-infected cells. (**F**) Cells were infected with UV inactivated RV-SA11. At 8hpi the total RNA was extracted and the relative expression of let-7g was quantified by qRT-PCR analysis. (**G**) VP6 RNA level was measured as a marker of viral infection in UV-inactivated RV-SA11 infected cells. Results are presented as the means and standard deviations from at least two independent experiments.

To determine, whether RV regulates mTOR by modulation of only miR-99b or there are some other mechanisms playing around, we analysed upstream regulators of mTOR^38^. Therefore, the expression of Rheb-GTP, TSC1 and TSC2 were analyzed in whole cell lysates of RV-SA11 infected HT29 cells by immunoblotting. Results revealed significant (2–3 fold) reduction in Rheb-GTP expression, whereas the levels of both TSC1 and TSC2 increased (~5–6 fold) in virus-infected cells compared to mock-infected controls (Fig. 3A). These results suggested that miR-99b may not be the sole regulator of mTOR during RV infection.

**Figure 4:**
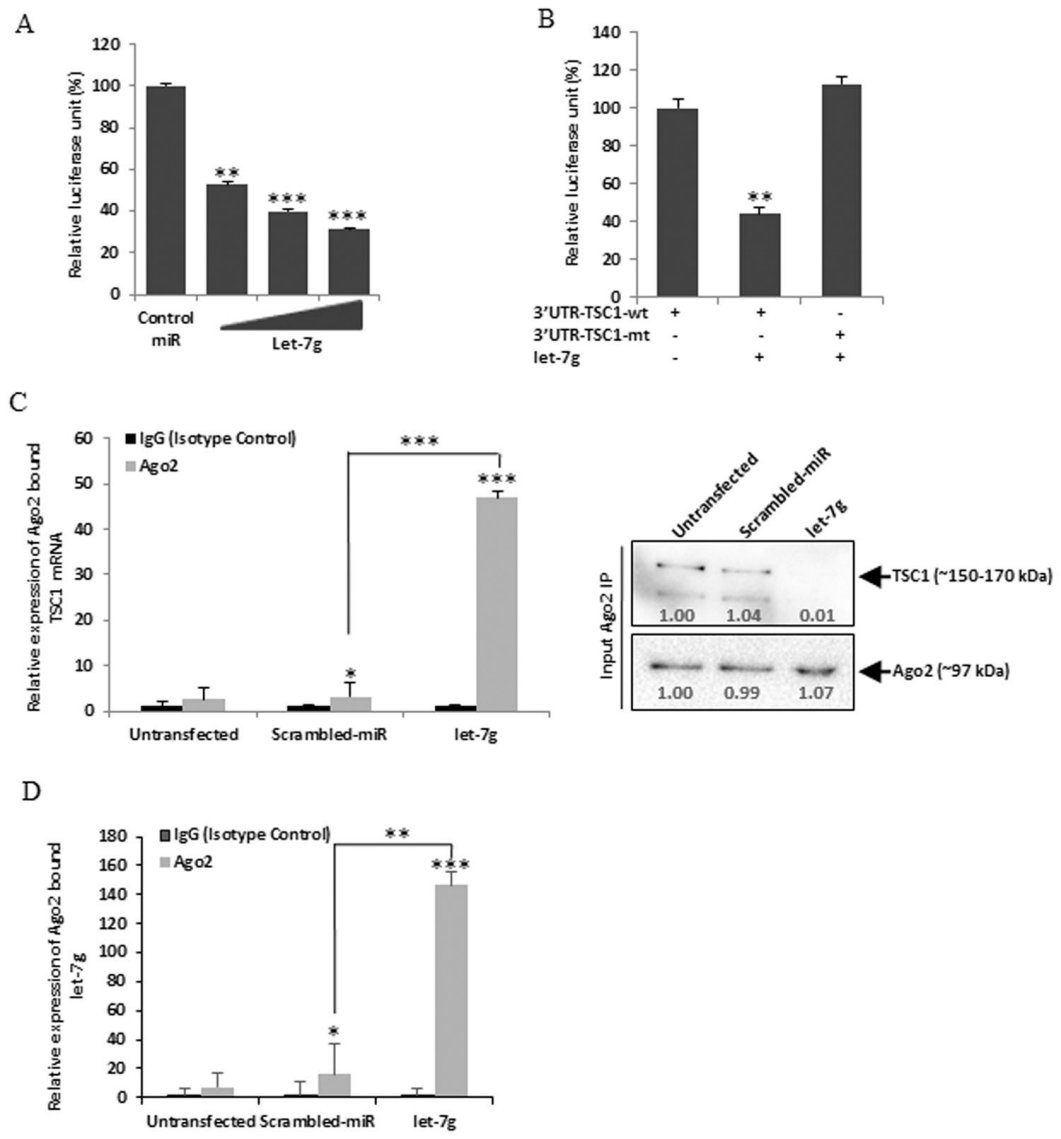
TSC1 is a direct target of let-7g. (**A**) 293 T cells were co-transfected with pMIR-REPORT luciferase construct containing the 3′UTR of TSC1 and different doses of the let-7g mimic (10, 20 or 40 nM). Relative luciferase activity was measured after 48 h of transfection. (**B**) Luciferase activity of the mutant 3′UTR of TSC1 was not altered in presence of mimic let-7g (40 nM) compared to the wild-type 3′UTR. (**C**) let-7g transfected HT29 cell lysates were immunoprecipitated with Ago2 specific monoclonal antibody or IgG2a isotype control. RNA was isolated from immunoprecipitates using RNeasy kit. let-7g bound TSC1 mRNA expression was analyzed by qRT-PCR (left panel). Expression of Ago2 and TSC1 were checked in each set of input sample lysates by immunoblotting (right panel). Relative expression was determined after normalization to control cells. (**D**) Relative expression of let-7g from Ago2 immunoprecipitates was analyzed by qRT-PCR. The results are shown as mean and standard deviation from representative of three technical replicates.

*In silico* analysis suggested the putative binding site of let-7g in the 3′UTR of TSC1 with a high mirSVR score (Fig. 3B). Moreover, let-7g is downregulated following RV infection as obtained in the miRNA microarray data (Supplementary Fig. S1A). In cells transfected with mimic let-7g, the level of TSC1 was significantly reduced (Fig. 3C). To ensure the biological processing of microRNA, let-7g precursor sequence was cloned in pmR-ZSGreen1 vector and it was overexpressed in HT29 cells (Fig. 3C right panel). Reduced expression of let-7g was also confirmed by qRT-PCR in HT29 cells infected with RV-SA11 (Fig. 3D) and MA104 cells infected with RV-SA11 and RV-KU (Supplementary Fig. S2A). The VP6 transcript was measured as a marker of RV infection in the same set of RNA (Figs 3E and S2B). No change in the expression of let-7g was found in HT29 cells infected with UV-inactivated RV-SA11 (Fig. 3F). Expression of VP6 RNA in UV-inactivated RV infected cells ensures the change in let-7g expression was virus-driven (Fig. 3G). The downregulation of let-7g infected with RV-SA11 and upregulation with InfA/PR8 in MDCK cells further confirmed the effect of RV infection on let-7g expression (Supplementary Fig. S1E). The mimiRNA algorithm showed the abundance (standard score of ~700 with p < 0.01) of let-7g in HT29 cells as well as in small intestinal tissues (Supplementary Fig. S2C). Therefore, these results suggest that RV infection significantly downregulates let-7g expression, whereas, TSC1 is a predicted target of let-7g. In order to validate the target, 3′UTR of TSC1 or its respective mutant fragment was cloned into pMIR-Report luciferase vector and co-transfected with mimic let-7g as described earlier. The reporter assay confirmed inhibition of 3′UTR-TSC1-wt expression following binding of mimic let-7g compared to the expression of 3′UTR-TSC1-mt (Fig. 4A,B). We further examined the association of let-7g with TSC1 mRNAs. Therefore, RNA immunoprecipitations with Ago2 specific monoclonal antibody or control IgG were performed to isolate TSC1 mRNA from let-7g overexpressed cells. The let-7g bound mRNA expression of TSC1 was significantly upregulated in Ago2 immunoprecipitates compared to the isotype control (Fig. 4C). Furthermore, Ago2 bound TSC1 mRNA level was increased in let-7g mimic transfected cells compared to scrambled-miR transfection (Fig. 4C). Similar results were obtained in the context of RV infection. Ectopic expression of let-7g mimic in RV infected cells restored the expression of Ago2 bound TSC1 mRNA level (Supplementary Fig. S2D). Ago2 and TSC1 expressions were analysed in the input lysates from each experimental set (Figs 4C and S2D). HT29 cells are difficult to transfect, therefore, the relative expression level of let-7g from the immunoprecipitates was analyzed by qRT-PCR (Fig. 4D). Together these reporter assays and Ago2 IP assays suggested that let-7g directly binds to the 3′UTR of TSC1.

### miR-99b and let-7g regulate autophagy during RV infection

**Figure 5:**
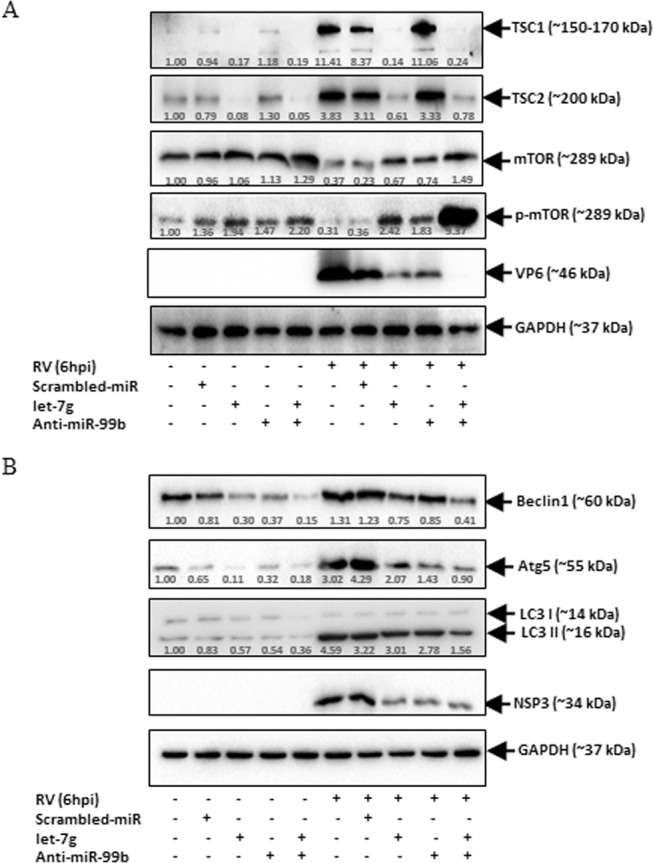
miR-99b and let-7g regulate autophagy during RV infection. HT29 cells were transfected with scrambled-miR, mimic let-7g, anti-miR-99b (40 nM each) or in combination (antagonist miRNA cocktail −50 nM total) and infected with RV-SA11. (**A**) The expressions of TSC1, TSC2, mTOR, and p-mTOR were examined by immunoblot analysis. GAPDH was used as an internal control for obtaining the expression levels after normalization. VP6 expression was checked to ensure virus infection. (**B**) Expressions of LC3I/II, Beclin1 and ATG5 were checked in the same cell lysates by immunoblotting. Normalization was done by GAPDH. Virus infection was confirmed by analysing NSP3 expression in the same cell lysates. Results are presented as the means and standard deviations from three experimental replicates.

Induction of autophagy during the early hours of RV infection is already reported^24^. Concurrent to the prior report, major autophagy markers such as Beclin1 and ATG5 expressions and LC3 lipidation were observed in RV infected cells compared to mock-infected controls (Supplementary Fig. S3A,B). Therefore, we hypothesized that RV infection-mediated suppression of let-7g and induction of miR-99b may induce autophagy through TSC1/2-mTOR pathway. To confirm our hypothesis, anti-miR-99b and mimic-let-7g were exogenously expressed either individually or in combination in HT29 cells followed by RV infection. Immunoblot analysis revealed suppression of TSC1-TSC2 expression and restoration of mTOR and p-mTOR in let-7g and/or anti-miR-99b transfected lysates compared to scrambled-miR transfected cells (Fig. 5A). The restoration of mTOR was higher in the cells where both let-7g and anti-miR-99b were co-transfected (Fig. 5A). mTOR is known as a negative regulator of autophagy signalling^37^. Therefore, we checked the expression of autophagy markers in the same cell extracts (Fig. 5B). The expression levels of LC3I/II, Beclin1 and ATG5 were significantly reduced in presence of mimic let-7g and anti-miR-99b in RV infected cells (Fig. 5B). To check whether transfection of the miRNA cocktail exerts any cellular toxicity or nonspecific interferon response, HT29 cells were transfected with mimic miRNAs or anti-miR or Poly I:C (25 µg/ml) control followed by cell viability assay and qRT-PCR for IFNα and IFNβ expression. The synthetic miRNA mimic or inhibitors used in this study did not exert any cellular toxicity or nonspecific induction of IFNα and IFNβ expression (Supplementary Fig. S3C,D). To nullify the super-physiological effect exerted by transfection of synthetic miRNAs, HT29 cells were transfected with pmR-ZsGreen1-pre-let-7g, followed by RV infection. In spite of biological processing of miRNA’s from pmR-ZsGreen1-pre-let-7g plasmid, a similar pattern of mTOR, p-mTOR, Beclin1, and ATG5 expressions were observed as in cells transfected with let-7g mimic (Supplementary Fig. S3E,F). Expression of let-7g in pmR-ZsGreen1-pre-let-7g transfected cell was checked by qRT-PCR (Supplementary Fig. S3G). Hence, the results indicate that RV modulates let-7g and miR-99b to regulate mTOR expression, therefore autophagy.

### Anti-miR-99b and let-7g limits RV replication

**Figure 6:**
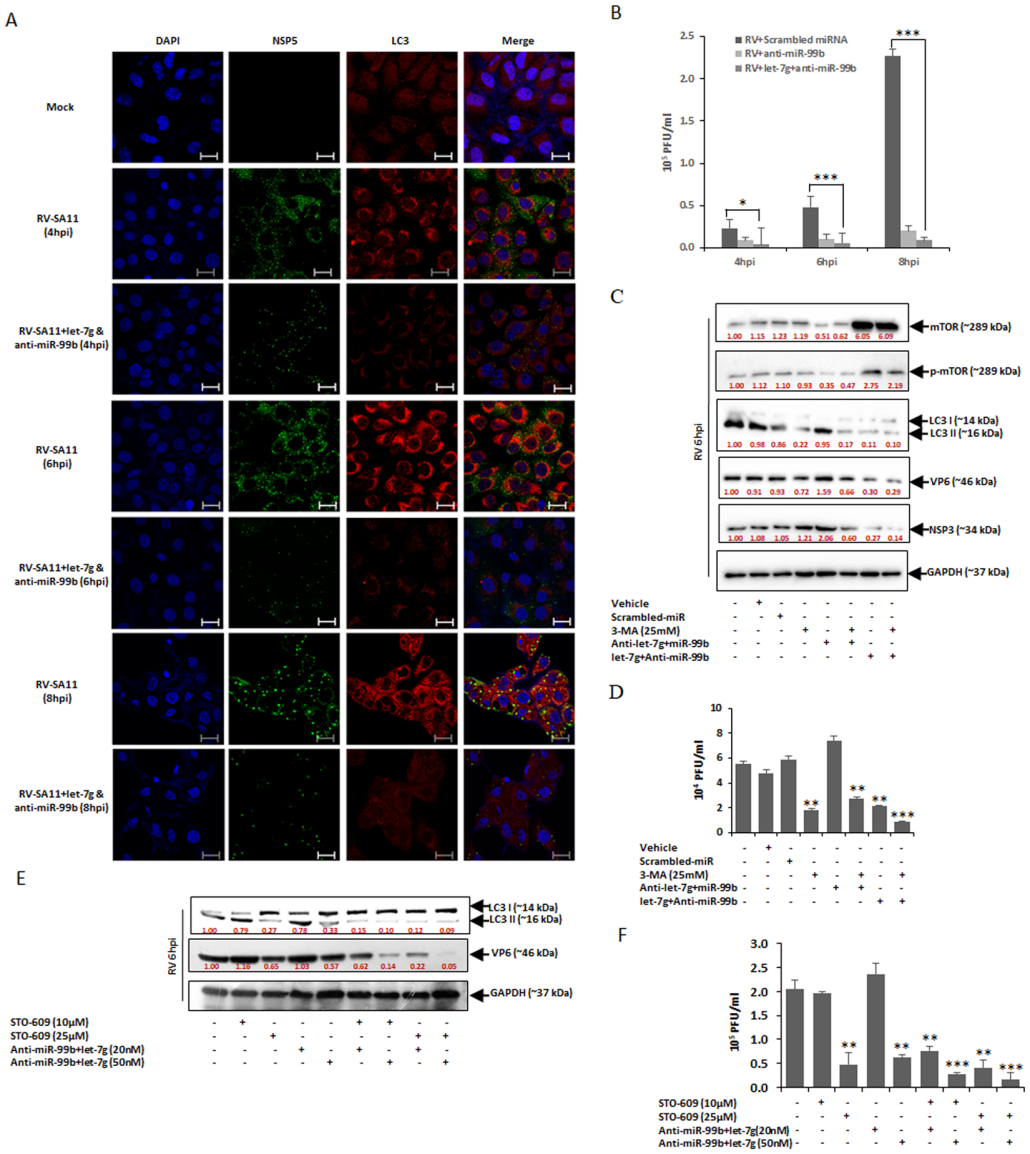
Ectopic expression of anti-miR-99b and let-7g blocks RV infection. (**A**) MA104 cells were transfected with anti-miR-99b and let-7g (50 nM) followed by RV-SA11 infection. Cells were then fixed, permeabilized at 4–8 hpi and stained with antibody against NSP5 to detect virus infection (green), endogenous LC3 (red). Scale bars: 20μm. (**B**) HT29 cells were transfected with control-miR, anti-miR-99b and/or mimic let-7g (50 nM) for 48 h followed by RV infection. Infectious virus particle was measured at indicated time points by virion quantification assay. (**C**) 3-MA treated HT29 cells were transfected with scrambled-miR, agonist or antagonist miRNA cocktail (50 nM) followed by infection with RV-SA11. The expression of mTOR, p-mTOR and LC3 lipidation were examined by immunoblot analysis. VP6 and NSP3 expressions were analysed in the same set of samples to ensure virus infection. (**D**) Infectious RV particle was measured by virion quantification assay in the same experimental conditions in presence of 3-MA at 6hpi. (**E**) HT29 cells were treated with STO-609 (10 µM and 25 µM) and/or antagonist miRNA cocktail (20 nM and 50 nM total concentration) in different combinations followed by immunoblotting using RV-VP6 specific antibody. (**F**) Virion quantification assay was done in the same experimental conditions for quantification of infectious RV particles. The results are presented as the means and standard deviations from at least three independent experiments.

Since autophagy is inhibited by the synergistic inflection of miR-99b and let-7g, their downstream effect on RV replication was examined. Reduction of LC3 puncta formation and suppression of RV infection in presence of anti-miR-99b and mimic let-7g cocktail was confirmed by confocal microscopy (Fig. 6A). Viroplasms were visualized in RV-SA11 infected MA104 cells (4–8 hpi) using anti-NSP5 antibody (green) (Fig. 6A, panel 2). In agreement with the previous reports, virus-infected cells showed significant numbers of LC3 puncta (red) (Fig. 6A, panel 3). However, the introduction of anti-miR-99b/let-7g cocktail in RV infected cells led to a reduction of both LC3-II and NSP5 expression suggesting suppression of autophagy and viral replication (Fig. 6A, row 3, 5 and 7). Presence of anti-miR-99b/let-7g cocktail in RV infected cells caused significant decrease in the number of cells showing intracellular LC3 dots (Supplementary Fig. S4A). Furthermore, HT29 cells were transfected with scrambled miR or anti-miR-99b or in combination with mimic let-7g, followed by RV-SA11 infection. Plaque assay was done and the level of viral VP6-RNA was examined to measure the infectious RV particle. The results showed that simultaneous knockdown of miR-99b and overexpression of let-7g significantly suppressed RV replication as evident at indicated time points (Figs 6B and S4B). Taken together, these results suggested that the combined effect of miR-99b knockdown and overexpression of let-7g has a antiviral role in controlling RV replication.

To substantiate that anti-miR-99b and let-7g exert their anti-rotaviral effects specifically by inducing the mTOR pathway leading to negative regulation of autophagy, HT29 cells were transfected with the combination of anti-let-7g and miR-99b (agonist miRNA cocktail) or anti-miR-99b and let-7g (antagonist miRNA cocktail) in presence or absence of a known autophagy inhibitor, 3-MA (3-Methyladenine) followed by RV-SA11 infection. Concurrently, anti-miR-99b and let-7g cocktail resulted in induction of mTOR and downregulation of autophagy marker LC3-II both in presence or absence of 3-MA (Fig. 6C; lanes 7, 8). However, in cells transfected with the agonist miRNA cocktail, RV-SA11 infection resulted in downregulation of mTOR and subsequent induction of autophagy marker LC3-II confirming the direct role of these miRNAs in modulating autophagy (Fig. 6C; lane 5). Treatment with 3-MA which exerts its anti-autophagy role downstream of mTOR resulted in inhibition of induction of autophagy marker LC3-II by anti-let-7g and miR-99b cocktail (Fig. 6C; lane 6). Similar effects of anti-let-7g and miR-99b cocktail were observed in plaque assay as an increase in viral titers were observed compared to significantly low viral titers in cells treated with anti-miR-99b and let-7g cocktail. As reported earlier, 3-MA treatment alone resulted in reduced viral titers, and the combination of 3-MA and miRNA cocktail resulted in > 90% reduction in viral replication (Fig. 6D).

To analyse whether previously described NSP4 mediated CaMKK-β signalling and microRNA mediated autophagy signalling act in parallel following virus infection, RV infected HT29 cells were treated with CaMKK-β inhibitor STO-609 (10 μM and 25 μM) and/or antagonist miRNA cocktail (20 nM and 50 nM) in different combination followed by assessing LC3 lipidation. Even though STO-609 (10 µM) or miRNA-cocktail (20 nM) had no effect on LC3 lipidation at low dosage (Fig. 6E; lane 2, 4), the combination of STO-609 (10 µM) and miRNA cocktail (20 nM) significantly suppresses LC3-II expression therefore LC3 lipidation (Fig. 6E; lane 6). Furthermore, plaque assay confirmed that STO-609 and cocktail of anti-miR-99b and let-7g combination synergistically suppress RV replication (Fig. 6F). Among many other regulators, PI3K-Akt signalling is one of the potent regulators of mTOR. Therefore, we analysed the expression of miR-99b and let-7g in presence of PI3K inhibitor LY294002 (5 μM) along with CaMKK-β inhibitor STO-609 treated RV infected cells to examine whether these two pathways have any involvement in RV driven dysregulation of these two miRNAs. Introduction of these inhibitors had no effect on cell viability or regulation of miR-99b or let-7g expression (Supplementary Fig. S4C,D). Overall, these results indicate that miR-99b and let-7g along with multiple other pathways act either synergistically or in parallel to modulate autophagy for successful RV replication.

## Discussion

Over the past few years, understanding the role of miRNAs in various physiological and disease conditions has become an intriguing field of study. Multiple miRNAs together with their multiple downstream target genes form a regulatory network to control several fundamental biological pathways. Emerging evidence suggests that cellular miRNAs play a vital role in the regulation of viral replication^39^. miR-204 and miR-4331 inhibit influenza A virus replication by targeting viral HA and NS gene, respectively^23^. miR-181c limits HCV replication by targeting HOXA1 as well as viral genome^12^. miR-122, miR-199a, and miR-210 also suppress HBV replication^40,41^. On the other hand, miR-21 acts as a positive regulator of dengue virus replication in HepG2 cells^42^. miR-30a-5p promotes replication of porcine circovirus type-II by enhancing autophagy^43^. During RV infection, differential miRNA expression was observed where one of the miRNA, miR-142–5p was shown to facilitate RV replication by targeting TGFβ signalling pathway^17^. *In silico* analysis of other differentially expressed miRNAs, miR-99b and let-7g predicted their role in regulating mTOR signalling. Interestingly, miR-99b was upregulated whereas let-7g was downregulated during RV infection. mTOR is a kinase, which serves as a component of two distinct protein complexes, mTOR complex 1 (mTORC1) and mTOR complex 2 (mTORC2), and regulates different cellular processes like cell growth, cell proliferation, cell motility and survival, protein synthesis and autophagy^44,45^. Autophagy has been reported to facilitate RV infection through NSP4 mediated activation of CaMKK-β signalling^24^. This study highlights a parallel pathway which modulates autophagy during RV infection through the synchronized expression of two miRNAs, miR-99b and let-7g, which in turn modulate TSC1/2-mTOR signalling pathway.

miR-99b is reported to be dysregulated in many pathophysiological conditions and miR-99b mediated mTOR regulation is also investigated in pancreatic cancer, colorectal cancer etc^46,47^. Recently it was found that miR-99b family regulates IGF1R-PI3K-Akt-mTOR signalling mediated autophagy to promote HBV infection^36^. As miR-99b was upregulated during RV infection, an association of miR-99b with mTOR was confirmed during RV infection (Figs 1 and 2). mTOR is a potent negative regulator of autophagy and RV promotes autophagosome formation for proper viral replication^24,34^. Moreover, this is such an important complex that host and pathogen constantly battle over mTOR to gain advantage^48^. Among many other cellular signalling pathways which regulate mTOR, PI3K-Akt signalling is the most studied one. Recent studies showed that RV promotes NSP1 dependent Akt phosphorylation and inhibition of PI3K-Akt results in reduced RV replication^49^. In spite of activation of PI3K-Akt pathway during early hour of RV infection, mTOR expression was found to be downregulated (during 4–8 hpi) in the present study (Fig. 2), followed by induction during later time points^50^. Moreover, upstream regulators of mTOR e.g. TSC1, TSC2 expressions were induced and Rheb-GTP was suppressed following RV infection (Fig. 3), which suggested additional pathway involved in regulation of TSC1, independent of NSP1 mediated PI3K-Akt signalling. let-7g, a microRNA which is downregulated following RV infection, was found to target TSC1 (Figs 3 and 4). Moreover, RV mediated decrease in let-7g expression led to a reduction in the Ago2 bound mRNA pool of TSC1 which was restored in presence of mimic let-7g (Supplementary Fig. S2D). The unaltered expression of Ago2 in the inputs suggest that changes in the expression of let-7g were entirely due to RV infection, not merely a nonspecific one. PI3K inhibitor treatment had no effect on the expression of let-7g as well as on miR-99b, suggesting PI3K-Akt independent regulation of these microRNAs (Supplementary Fig. S4D).

In the present study, we found that RV mediated downregulation of let-7g and upregulation of miR-99b ultimately suppresses mTOR expression following RV infection. Ectopic expression of anti-miR-99b and mimic let-7g resulted in suppression of TSC1/2 and restoration of mTOR during early hour of RV infection (Fig. 5). Furthermore, this study also identified significant inhibition of autophagy markers such as LC3 lipidation, Beclin1 and ATG5 in presence of anti-miR-99b and mimic let-7g during RV infection (Fig. 5). Therefore, in agreement with the previous study^24,34^, we observed a positive correlation between autophagy and RV replication during early phases of infection. In addition, consistent to our findings, a recent study showed that Rotavirus-encoded small RNA, RV-vsRNA1755, suppresses the PI3K/Akt pathway by targeting IGF1R, therefore triggering autophagy during early stage (<8hpi) of RV infection^51^. In contrast to our present study, Yin Y *et*. *al*., reported that mTOR inhibition exerted 4E-BP1 mediated induction of autophagy that negatively influences RV infection in CaCo2 cells at 48-hour post infection^52^. This discrepancy could result from downregulation of mTOR by shRNA transfection (48 h) prior to RV infection which may have a negative effect on viral entry or initiation of replication. It can also be hypothesized the time point dependent dynamics of mTOR expression and autophagy regulation by RV during early hours of infection that ensures proper viral replication.

miRNAs are well known for targeting hundreds of cellular mRNAs, creating major confusion regarding the specificity of a certain miRNA on a particular cellular signalling pathway. Even though the ectopic expression of mimic miR-99b and anti-let-7g could regulate the expression of mTOR, but they failed to induce autophagy and RV infection in presence of 3-MA (inhibitor of autophagosome formation) (Fig. 6C,D), confirming the specificity of these microRNAs on mTOR mediated autophagy following RV infection.

**Figure 7:**
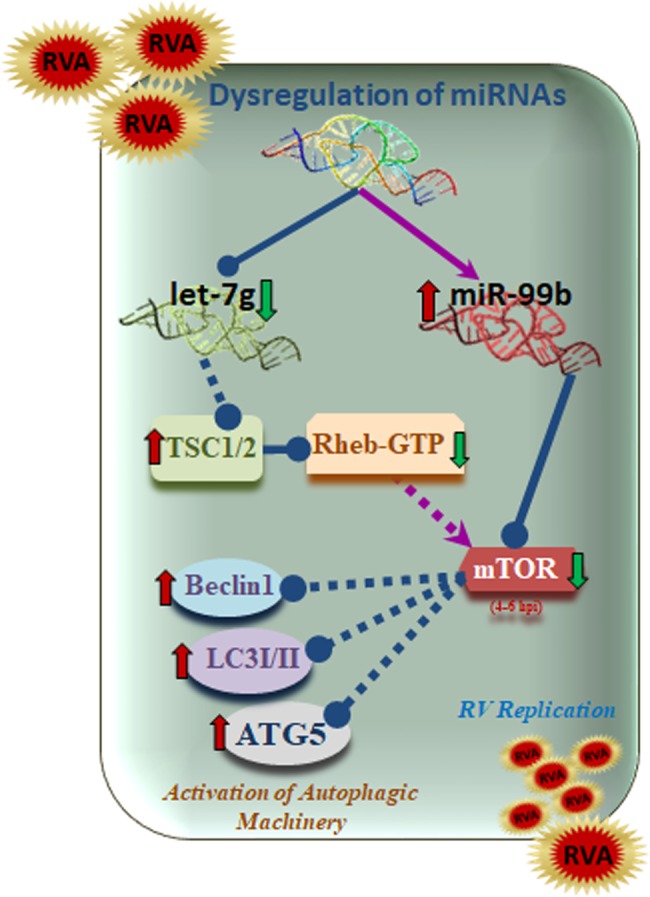
Schematic diagram showing the crosstalk between miR-99b, let-7g, and RV. RV infection downregulates let-7g which in turn elevates TSC1 expression. Inhibition of let-7g also promotes TSC1 mediated suppression of Rheb-GTP expression. On the other hand, RV infection upregulates miR-99b that directly targets mTOR. RV mediated upregulation of miR-99b and suppression of Rheb-GTP leads towards containment of mTOR expression. These results lead towards activation of autophagy mediators during RV infection. Arrows denote upregulation, and blunt arrows denote inhibition.

In summary, the results demonstrate a successful orchestration of two cellular miRNAs in modulating autophagy by targeting TSC1/2-mTOR signalling pathway, resulting in effective RV replication (Fig. 7). The combined effect of let-7g overexpression and miR-99b silencing results in significant inhibition of autophagy as well as viral replication as shown by reduced viral titre. Confocal microscopy experiments also confirmed reduced LC3II and RV-NSP5 expression in presence of antagonist miRNA cocktail. Hence, the study highlights the importance of combination therapy to simultaneously block multiple miRNAs, which may provide new insights on the development of antiviral therapeutic strategy against viral infections.

## Materials and Methods

### Cells and viruses

Human colorectal adenocarcinoma cells (HT29), human embryonic kidney cell (293 T), monkey kidney epithelial cells (MA104) and canine kidney epithelial cells (MDCK) were maintained at 37 °C, 5% CO_2_ either in DMEM (Dulbecco’s Modified Eagle’s Medium) or MEM (Minimum Essential Medium) supplemented with 10% heat-inactivated FBS (fetal bovine serum), 2mM L-glutamine, 2 mM sodium pyruvate and 1X penicillin, streptomycin, and fungizone. The RV strain SA11 (H96) and KU and influenza A strain PR8 has been used for this study. For infection, the virus was activated with acetylated trypsin (10 g/ml) at 37 °C for 30 minutes. Cells were incubated with the preactivated virus of 2 moi (multiplicity of infection) for 45 min at 37 °C. After adsorption and removal of the viral inoculum, serum-starved media has been used for continuation of infection. For UV inactivation, the virus was pretreated with 40 μg/ml psoralen AMT (prepared from 1 mg/ml stock solution in 50% ethanol and 50% water) for 15 minutes and then irradiated by long-wave UV-light (365 nm) for 2 hours under ice cold condition^53^. Time of virus removal was 0hpi. Extracted and purified viral preparations were titrated and calculated by plaque assay following the formula: moi = plaque-forming units of virus used for infection/number of cells^53,54^.

### Cell viability assay

HT29 cells were transfected with control miRNA or mimic or inhibitor of miR-99b and let-7g or miRNA cocktail or small-molecule inhibitors STO-609, LY294002, and 3-MA in 96 well plates. At 48 h post-transfection, cells were treated with 20 μl of the reagent solution in the 100 μl serum-free medium for 4 h at 37 °C in a humidified 5% CO_2_ atmosphere, as described by Cell titer 96® Aqueous One Solution Cell Proliferation Assay kit (Promega: G3581). The cell plate was measured spectrophotometrically at 490 nm in Varioskan Multimode Reader (Thermo Fisher Scientific). The absorbance was directly proportional to the number of living cells in culture. The percent viability was calculated considering 100% viability for mock control at similar endpoints.

### RNA quantitation and reverse transcription-quantitative PCR (qRT-PCR)

Total RNA was isolated using TRIzol reagent (Invitrogen: 15596018). cDNA was synthesized by using miR-99b, let-7g or U6-specific primers with a TaqMan microRNA reverse transcription kit (Applied Biosystem: 4366597) and a random hexamer with Superscript III reverse transcriptase (Invitrogen: 18080-051) for mTOR, TSC1, RV-VP6, and GAPDH. Real-time PCR was performed using TaqMan universal PCR master mix and 6-carboxyfluorescein (FAM)-MGB probes (Applied Biosystem) for quantification of miR-99b (assay identification number 000436) and let-7g (assay identification number 002282). U6 (assay identification number 001973) was used as an endogenous control for microRNA expression. For gene expression studies, TaqMan universal PCR master mix and FAM-MGB probes for IFNα (assay identification number Hs00353738) and IFNβ (assay identification number Hs01077958) and SYBR® Green master mix (Applied Biosystems, USA) with specific primers for mTOR, TSC1, and RV-VP6, were used for quantitation (Supplementary Table). GAPDH was used as an endogenous control for gene expression studies. The relative expression levels were normalized to the endogenous control by using the 2^−ΔΔCT^ formula (ΔΔC_T_ = ΔC_T_ of the sample −ΔC_T_ of the untreated control).

### Luciferase reporter assays

The 3′UTR luciferase reporter constructs of mTOR and TSC1 were generated by cloning the PCR-amplified human mTOR and TSC1 mRNA 3′UTRs (miR-99b and let-7g target sites, respectively) into the MluI/HindIII site of the pMIR-REPORT miRNA expression luciferase reporter plasmid (Ambion: AM5795). Mutant 3′UTRs of mTOR or TSC1 were used as a control in parallel. 293 T cells were co-transfected, using Lipofectamine^®^ RNAiMAX Reagent (Thermo Fischer Scientific: 13778150), with the luciferase reporter plasmid and control-miR or different doses of mimic miR-99b or let-7g and firefly luciferase activity was determined by Dual-Luciferase^®^ Reporter Assay System (Promega: E1960) after normalization to the expression of control Renilla luciferase. Primers used for the mTOR and TSC1 3′UTRs are listed in Supplementary Table.

### Cloning and transfection

Complementary strands of pre-let-7g were obtained from UCSC genome browser along with approximately 100 bp of upstream and downstream flanking region; amplified and ligated into XhoI/KpnI site of pmR-ZsGreen1 mammalian expression vector designed to constitutively express microRNA of interest followed by transfection in HT29 cells with Lipofectamine 2000 (Thermo Fischer Scientific: 11668019). Mimics and inhibitors against hsa-miR-99b and hsa-let-7g were obtained from Thermo Fischer Scientific. Mimics and inhibitors of specified miRNAs were transfected (final concentration 20–50 nM unless stated otherwise) in 293 T and HT29 cells with Lipofectamine® RNAiMAX Reagent, according to manufacturer’s recommendations.

### Immunoblot analysis

Cell lysates were subjected to polyacrylamide gel electrophoresis and transferred onto Polyvinylidene difluoride (PVDF) membranes. The membranes were blocked with 5% non-fat dried milk and incubated with specific antibodies. The membranes were probed with antibodies to mTOR (#2972), p-mTOR (#2971), TSC1 (#4906), Rheb-GTP (#13879), LC3I/II (#12741), Beclin1 (#3495), ATG5 (#12994) (Cell Signaling), TSC2 (#sc-893) (Santa Cruz, USA) RV-VP6 (HyTest: 3C10) or RV-NSP3 (kind gift from Prof. Koki Taniguchi). Proteins were detected with Horseradish Peroxidase (HRP)-conjugated secondary antibodies (Pierce, USA) and enhanced chemiluminescence (ECL) substrate (Millipore, USA). The membranes were reprobed with GAPDH (Santa Cruz: sc-25778) as an internal control. Densitometry was performed using Image J. The gels/blots used in this manuscript were checked for their compliance with the digital image and integrity policies.

### Confocal Microscopy

Cells were grown on glass coverslips (18-mm square, no. 1; Blue Star) and transfected with anti-miR-99b and mimic let-7g in combination using Lipofectamine^®^ RNAiMAX Reagent. At 24 h post-transfection, cells were infected with RV-SA11 strain. Cells were then fixed in ice-cold 100% methanol for 15 min at −20 °C followed by permeabilization with PBS containing 0.1% Triton X-100 for 20 min at 4 °C. Nonspecific antibody sites were blocked with PBS containing 2% BSA (w/v) for 30 min at room temperature. For staining, cells were incubated with primary antibodies against LC3-I/II (1:200; Mouse monoclonal; Santa Cruz), and RV-NSP5 (1:200; Rabbit monoclonal; a kind gift from Prof. Koki Taniguchi) diluted in blocking solution, for overnight at 4 °C. Cells were then treated with DyLight 488-labeled goat-anti-rabbit (#35552) and Rhodamine-conjugated goat-anti-mouse (#31660) (Thermo Fisher Scientific) secondary antibodies for 30 min at room temperature. Nuclei were visualized after incubation with Vectashield containing 4′, 6′-diamidino-2-phenylindole (DAPI), and mounted on microscope slides. Mounted slides were observed on Olympus FV1200 (100X oil immersion) confocal microscope (Tokyo, Japan). The images were captured and processed using FLUOVIEW Viewer FV10-ASW v4.2 (Olympus Corp., Tokyo, Japan) and saved as 12-bit tagged TIFF images in RGB-format. For comparison between different samples, images were collected during a single session at identical excitation and detection settings.

### Ago2-RNA co-immunoprecipitation

HT29 cells were transfected with control-miR, mimic miR-99b or let-7g and lysed with lysis buffer [150 mM KCl, 25 mM Tris-HCl (pH 7.4), 5 mM EDTA, 1% Triton X-100, 5 mM DTT, protease inhibitor mixture, and 100 U/mL RNaseOUT (Invitrogen)]. Cell lysates were measured and clarified into two equal part. One part was kept aside as Input lysates and the other part was incubated with anti-Ago2 mAb (Cell Signaling: 2897) or isotype control IgG2a at 4 °C for overnight. Next day antibody coupled lysates were mixed with Protein G Sepharose beads (GE Healthcare: 17061802) for 4 h. The beads were washed four times and RNA was isolated using the RNeasy Mini Kit (Qiagen: 74104). mTOR and TSC1 expressions were quantified by qRT-PCR using SYBR^®^Green chemistry and miRNA expressions by TaqMan probe chemistry as described earlier. For Ago2 IP in RV infected samples, the HT29 cells were transfected with anti-miR-99b or mimic let-7g for 24 h followed by infection with RV-SA11 and RNA immunoprecipitation as described above.

### Statistical analysis

All results are presented as means from at least two or three independent experiments ± standard deviations. Statistical significance of the data has been analyzed using Student’s t-test. The figures having more than two experimental groups were analyzed by pairwise ANOVA using GraphPad Prism 7.0. P value of < 0.05 was considered to be statistically significant and all data were presented as asterisks (*p ≤ 0.05; **p ≤ 0.01; ***p ≤ 0.001).

## Supplementary information

Supplementary Figure and Table
