# Supplementary material for: Synchronized Orchestration of miR-99b and let-7g Positively Regulates Rotavirus Infection by Modulating Autophagy
Source: Sci Rep. 2019 Feb 4;9:1318. doi: 10.1038/s41598-018-38473-8 (PMC6362297; doi:10.1038/s41598-018-38473-8)
Supplement: Supplementary file 1 — Supplementary Figure and Table [file 41598_2018_38473_MOESM1_ESM.pdf]

Synchronized Orchestration of miR-99b and let-7g Positively Regulates Rotavirus Infection by Modulating Autophagy

Urbi Mukhopadhyay[a], Shampa Chanda[a], Upayan Patra[a], Arpita Mukherjee[a], Santanu Rana[b], Anupam Mukherjee[a§], Mamta Chawla-Sarkar[a§]

[a]Division of Virology, National Institute of Cholera and Enteric Diseases, Kolkata, WB, India; [b]Department of Zoology, University of Calcutta, Kolkata, WB, India.

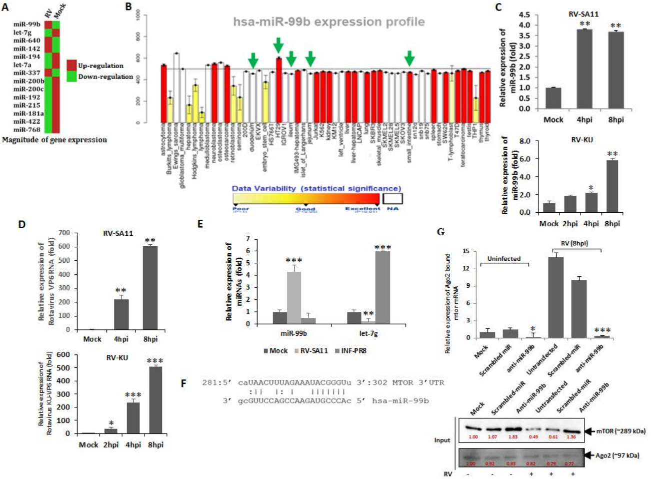

Supplementary Figure S1: (A) Representative heat map image of differential expression of cellular miRNAs in rotavirus infected cells compared to mock infected control cells. **(B)** mimiRNA screenshot: expression profile of hsa-miR-99b across multiple tissue types and cells (cropped and reduced). The expression levels transformed into a standard score spanning 1–1000 (*y*-axis) was derived from multiple experiments (*x*-axis). Histograms represent the mean of this score for all experiments and error bars represent standard error of the mean. The variability of the data collected is shown by colour shading for each tissue or cell type. The green arrows denote the cell line and the tissues of our interest. **(C)** Relative expression of miR-99b in MA104 cells infected with either RV-SA11 (upper panel) or RV-KU (lower panel) quantified by qRT-PCR. **(D)** Relative expression of VP6 RNA in the same samples measured by qRT-PCR. **(E)** Relative expression of miR-99b (left panel) and let-7g (right panel) in MDCK cells infected with either RV-SA11 or INFa-PR8 quantified by qRT-PCR. **(F)** Schematic diagram showing putative binding site of miR-99b in the 3'UTR of mTOR mRNA. **(G)** HT29 cells were kept untransfected or transfected with scrambled miR or anti-miR-99b followed by mock infection or RV-SA11 infection and Ago2 RIP was performed. Ago2 bound mtor mRNA expressions were analysed by qRT-PCR. Expressions of mTOR and Ago2 were checked in the input lysates. The results are shown as mean and standard deviation from representative of two technical replicates.

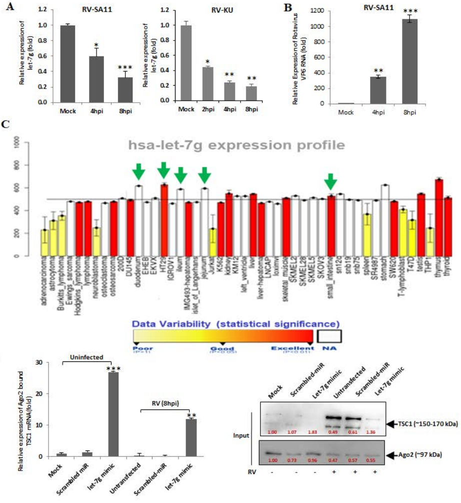

Supplementary Figure S2: (A) Relative expression of let-7g in MA104 cells infected with RV-SA11 (left panel) and RV-KU (right panel) quantified by qRT-PCR. **(B)** Expression of RV-VP6 checked in the same samples by qRT-PCR. **(C)** mimiRNA screenshot: expression profiles of hsa-let-7g across multiple tissue types and cells (cropped and reduced). The expression levels transformed into a standard score spanning 1–1000 (*y*-axis) was derived from multiple experiments (*x*-axis). Histograms represent the mean of this score for all experiments and error bars represent standard error of the mean. The variability of the data collected is shown by colour shading for each tissue or cell type. The green arrows denote the cell line and the tissues of our interest. **(D)** HT29 cells were kept untransfected or transfected with scrambled miR or mimic let-7g followed by mock infection or RV-SA11 infection and Ago2 RIP was performed. Ago2 bound TSC1 mRNA expressions were analysed by qRT-PCR. Expressions of TSC1 and Ago2 were checked in the input lysates. The results are shown as mean and standard deviation from representative of at least two technical replicates.

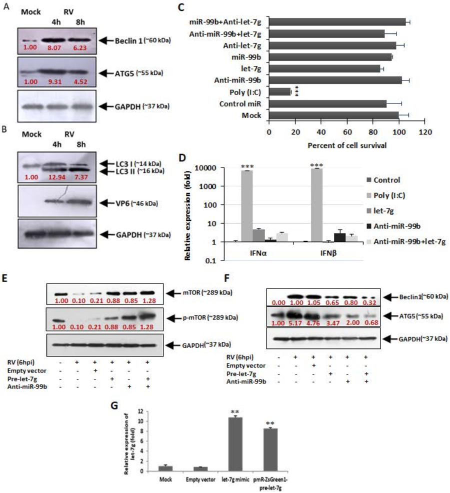

Supplementary Figure S3: Relative expression of **(A)** Beclin 1 and ATG5 and **(B)** LC3II lipidation and RV infection in SA11 infected HT29 cells quantified by immunoblot analysis using specific antibodies, respectively. GAPDH was used for normalization and VP6 was used for checking virus infection. **(C)** HT29 were transfected with scrambled-miR or mimics and inhibitors of let-7g or miR-99b either singly or in combinations. Synthetic Poly (I:C) (25 µg/ml) was used as control. Viable cells were measured by using Cell titer 96® Aqueous One Solution Cell Proliferation assay kit at 48h post transfection. Data are presented as the means and standard deviations from two independent experiments. **(D)** HT29 cells were transfected with mimic let-7g along or/and anti-miR-99b followed by quantitative PCR for IFNα and IFNß. Poly(I:C) treated cells were taken as positive inducer of IFNs production. HT29 cells were transfected with anti-miR-99b alone or in combination with pmR-ZsGreen1-pre-let-7g followed by RV-SA11 infection. Western blot analysis was done for **(E)** mTOR, p-mTOR and **(F)** Beclin1 and ATG5. **(G)** Relative expression of let-7g in mimic transfected and pre-miRNA sequences containing pmR-ZsGreen1 transfected HT29 cells quantified by qRT-PCR analysis. The results are shown as mean and standard deviation from representative of at least two technical replicates.

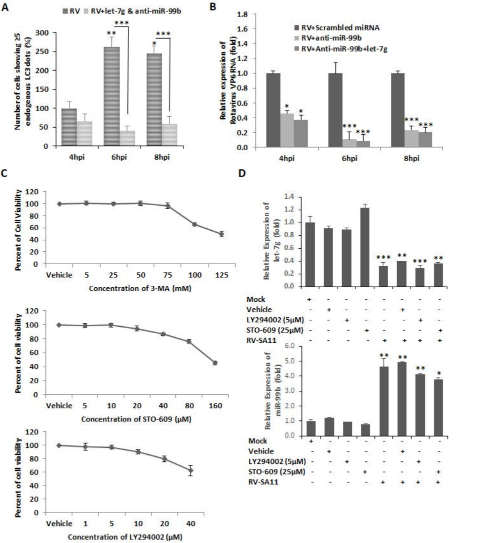

Supplementary Figure S4: **(A)** 250 cells per experimental condition were examined under fluorescence microscope and number of LC3 dots were scored in a blinded fashion. **(B)** RNA was isolated and RV-VP6 mRNA expression was analyzed by qRT-PCR at different time points. **(C)** HT29 cells were treated with autophagy inhibitor, 3-MA, PI3K inhibitor LY294002 or CaMKK-β inhibitor STO-609 and viable cells were measured at 48h post treatment. **(D)** HT29 cells were untreated or treated with vehicle or PI3K inhibitor LY294002 (5μM) or CaMKK-β inhibitor STO-609 (25μM) followed by mock infection or infection with RV-SA11(8hpi). Total RNA was isolated and qRT-PCR was done to check the expression of let-7g (upper panel) and miR-99b(lower panel). Results are shown as mean and standard deviation from representative of three technical replicates.

Supplement Table: Sequences of different primers used in the study

No.	Primer	5'<-----Sequence----->3'
1	mTOR F	GCT GTG AGG TCT GAG TTT AAG G
2	mTOR R	ATT GCC TTC TGC CTC TTA TGG
3	TSC1 F	GCT ATG GGT GTG CTA CTT CTA C
4	TSC1 R	AGC TTG TGG TGG TTC AGT TAT
5	VP6 F	GCA CAG CCA TTC GAA CAT CAT GC
6	VP6 R	TGC ATC GGC GAG TAC AGA CTC
7	GAPDH F	AAT CCC ATC ACC ATC TTC CAG
8	GAPDH R	AAA TGA GCC CCA GCC TTC
9	mTOR-wt_3'UTR_F	ACA CAA CGC GTG ATT TGG TTC CCA GGA CA
10	mTOR_3'UTR_R	AGC AAA GCT TTC ATT CTT CCA TCA GCA AG
11	mTOR-mt_3'UTR_R	TCA AAA GCT TTA TTT CTA AAG TTA TGG ATC T
12	TSC1-wt_3'UTR_F	TCT GAT ACG CGT CCT AGA AAG TTC TGT GTA
13	TSC1-mt_3'UTR_F	CTT GTA CGC GTT TCC CCC ACT ACC TCT TAT T
14	TSC1_3'UTR_R	CAG TGA AGC TTC CTT CCC TCC TAT GGA GAA
15	pre-let-7g_Xho1_F	CTC GAG AGC TTT GCT GCC AAG CCT CTG CTG T
16	pre- let-7g _Kpn1_R	GGT ACC CCT AAG AAG AAA AAG ACT TCC TCC CC
